# Dissemination of Carbapenemases (KPC, NDM, OXA-48, IMP, and VIM) Among Carbapenem-Resistant *Enterobacteriaceae* Isolated From Adult and Children Patients in China

**DOI:** 10.3389/fcimb.2020.00314

**Published:** 2020-07-03

**Authors:** Renru Han, Qingyu Shi, Shi Wu, Dandan Yin, Mingjia Peng, Dong Dong, Yonggui Zheng, Yan Guo, Rong Zhang, Fupin Hu, China Antimicrobial Surveillance Network (CHINET) Study Group

**Affiliations:** ^1^Institute of Antibiotics, Huashan Hospital, Fudan University, Shanghai, China; ^2^Key Laboratory of Clinical Pharmacology of Antibiotics, Ministry of Health, Shanghai, China; ^3^Department of Clinical Laboratory, School of Medicine, Second Affiliated Hospital of Zhejiang University, Hangzhou, China

**Keywords:** carbapenem-resistant *Enterobacteriaceae*, *bla*_KPC-2_, *bla*_NDM_, *bla*_OXA-48-like_, *bla*_IMP_

## Abstract

This study aimed to investigate the dissemination and characteristics of *bla*_KPC_, *bla*_NDM_, *bla*_OXA-48-*like*_, *bla*_IMP_, and *bla*_VIM_ among the carbapenem-resistant *Enterobacteriaceae* (CRE) strains isolated from adult and children patients. A total of 935 non-duplicate CRE strains were collected from 36 hospitals in 24 provinces or cities across China from 2016 to 2018. Antimicrobial susceptibility testing was performed by broth microdilution method and carbapenemase genes *bla*_KPC_, *bla*_NDM_, *bla*_OXA-48-*like*_, *bla*_IMP_, and *bla*_VIM_ were screened by PCR and confirmed by DNA sequencing. Overall, carbapenemases were produced in 97.4% (911/935) of CRE strains, including KPC-2 (51.6%, 482/935), NDM (35.7%, 334/935), and OXA-48-like carbapenemases (7.3%, 68/935). Overall, the most prevalent carbapenemase gene was *bla*_KPC-2_ among *Klebsiella pneumoniae* (64.6%, 457/709) and the CRE strains isolated from adult patients (70.3%, 307/437), and *bla*_NDM_ among *Escherichia coli* (96.0%, 143/149) and the CRE strains from children (49.0%, 247/498). The *bla*_OXA-232_-positive carbapenem-resistant *K. pneumoniae* (9.3%, 66/709) were all isolated from children. Sixteen strains were positive for *bla*_IMP_ and 9 strains produced multiple carbapenemases. No strain was positive for *bla*_VIM_. Most of the CRE strains (>90%) were resistant to cephalosporins and carbapenems, more than half (>50%) were resistant to aminoglycosides and fluoroquinolones, but the majority (95.8 and 98.4%) were susceptible to polymyxin B and tigecycline. Ceftazidime-avibactam showed excellent *in vitro* activity against *bla*_KPC-2_ and *bla*_OXA-48-*like*_ positive strains (100% susceptible). In China, KPC-2, NDM, and OXA-48-like carbapenemases were predominant among CRE clinical isolates. The most prevalent carbapenemase gene was *bla*_KPC-2_ among *K. pneumoniae* isolates from adult patients, and *bla*_NDM_ among *E. coli* isolates from children.

## Introduction

*Enterobacteriaceae* are opportunistic pathogens causing severe hospital-acquired infections (Feil, 2016). The spread of carbapenemase-producing *Enterobacteriaceae* (CPE) has been a global threat to public health. Carbapenems have conventionally been used for treating infections caused by extended-spectrum β-lactamase-producing *Escherichia coli* and *Klebsiella pneumoniae*, and are still considered as last resort antibiotics to date (van Duin and Doi, 2016). According to the data from China Antimicrobial Surveillance Network (CHINET, www.chinets.com), the resistance rate of *K. pneumoniae* to meropenem and imipenem rapidly increased from 2.9 and 3.0% in 2005 to 26.3 and 25% in 2018, respectively. In Europe, carbapenem-resistant *K. pneumoniae* are most widespread in the Mediterranean and Balkan countries with a prevalence of 60% in Greece and 40% in Italy, respectively (Perez and Villegas, 2015; Feil, 2016). The production of carbapenemases including KPC, NDM, and OXA-48-like is the most common resistance mechanism among carbapenem-resistant *Enterobacteriaceae* clinical isolates (Nordmann et al., 2012; Goodman et al., 2016). The *bla*_KPC_-positive *Enterobacteriaceae* were widespread in the United States, Latin America, Italy, Greece, the Middle East, and China (Albiger et al., 2015; Feil, 2016; Villegas et al., 2016; Iovleva and Doi, 2017). The *bla*_NDM_-positive *Enterobacteriaceae* were widespread in India, Pakistan, Bangladesh, Italy, Poland, Denmark, Latin America, and African countries (Yong et al., 2009; Albiger et al., 2015; van Duin and Doi, 2016). The *bla*_OXA−48−like_-positive strains remained rare in the US, in contrast to the prevalence in Turkey, Spain, France, Belgium, Romania, Middle East, Africa, Asia, and South America as well (Albiger et al., 2015). These infections are usually associated with very poor prognosis and high mortality, especially in neonates or high-risk immunocompromised patients (Falagas et al., 2014; Feil, 2016). In China, the presence of *bla*_KPC_ and *bla*_NDM_ is responsible for phenotypic resistance in most of the CRE strains (Zhang et al., 2017; Wang et al., 2018). Most researches currently focus on the dissemination of carbapenemases among CRE strains isolated from adult patients, while only a few are available to investigate the distribution of carbapenemases among CRE strains isolated from children. To obtain the comprehensive characteristic of carbapenemases among CRE isolated from both adults and children patients in China, we conducted this study to characterize the dissemination and characteristics of carbapenemases (including KPC, NDM OXA-48, IMP, and VIM) among CRE clinical isolates and the susceptibility to antimicrobial agents.

## Materials and Methods

### Clinical Strains

**Figure 1:**
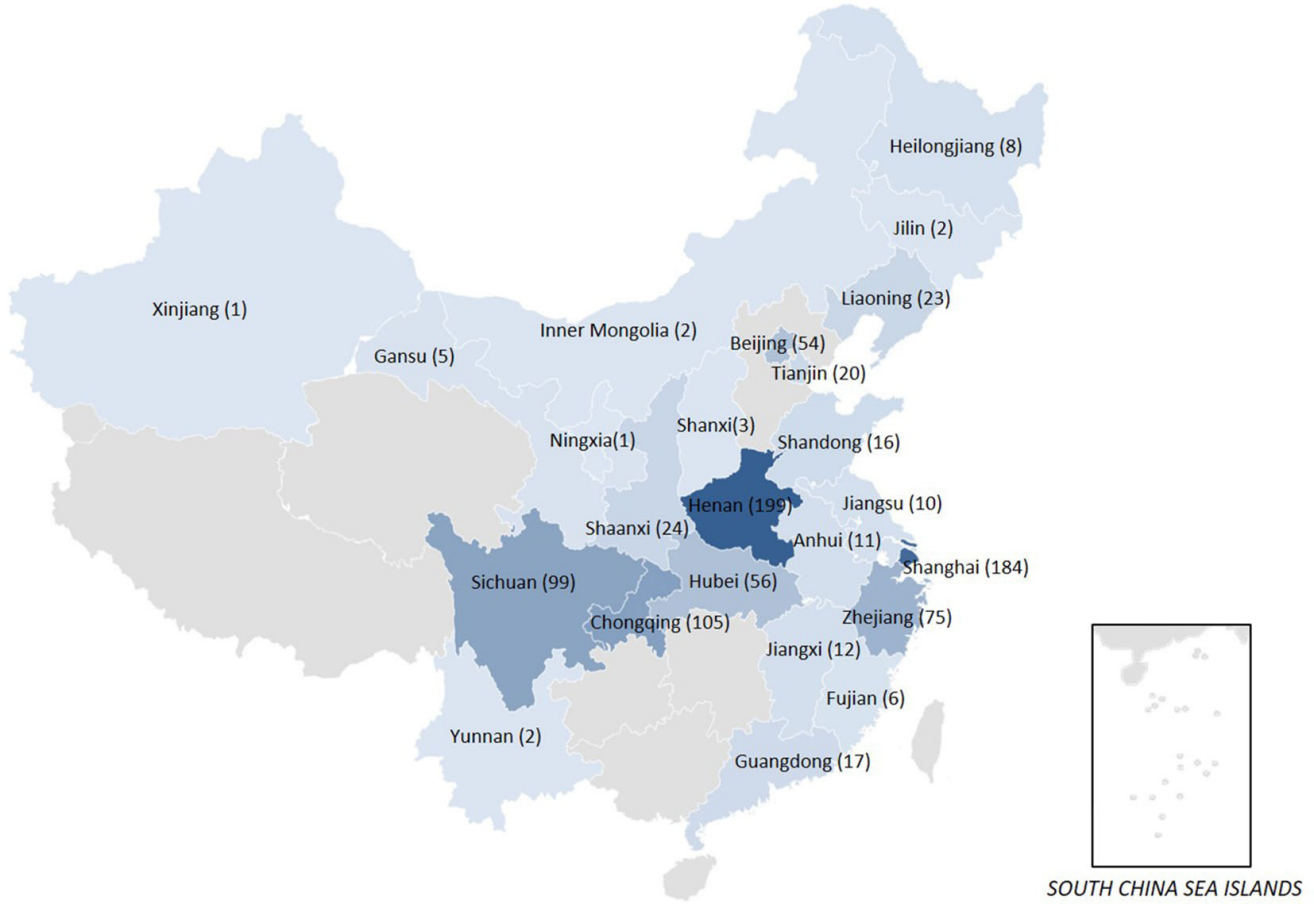
The map of CRE clinical strains collected from 24 provinces or cities in China.

From January 2016 to December 2018, a total of 935 non-duplicate sequential CRE strains were collected from 36 hospitals in 24 provinces or cities across China (Figure 1), including *K. pneumoniae* (*n* = 709, 75.8%), *E. coli* (*n* = 149, 15.9%), *Enterobacter cloacae* (*n* = 36, 3.9%), *Citrobacter freundii* (*n* = 14, 1.5%), *Serratia marcescens* (*n* = 8, 0.9%), *Enterobacter aerogenes* (*n* = 7, 0.7%), *Klebsiella oxytoca* (*n* = 7, 0.7%), *Morganella morganii* (*n* = 3, 0.3%), *Proteus vulgaris* (*n* = 1, 0.1%), *Providencia rettgeri* (*n* = 1, 0.1%). In this study, 46.7% (437/935) of CRE strains were collected from adult patients and 53.3% (498/935) from children patients. The *Enterobacteriaceae* strains resistant to at least one of the carbapenem antibiotics (ertapenem, meropenem, doripenem, or imipenem) or producing a carbapenemase (an enzyme that can make them resistant to carbapenem antibiotics) were defined as CRE by Centers for Disease Control and Prevention of USA (https://www.cdc.gov/hai/organisms/cre/technical-info.html#Definition). These CRE strains were isolated from sputum (27.5%), blood (27.1%), urine (17.0%), secreta (6.9%), bile (5.0%), ascites (3.2%), catheter (2.8%), drainage (2.8%), pus (1.4%) and other aseptic body fluid (6.4%). Species identification was confirmed by MALDI-TOF/MS system (bioMérieux, France). *E. coli* ATCC 25922, *E. coli* ATCC 35218, and *K. pneumoniae* ATCC 700603 were tested as the quality control strains for antimicrobial susceptibility testing.

### Antimicrobial Susceptibility Testing (AST)

AST was performed by the broth microdilution method recommended by the Clinical and Laboratory Standards Institute. Minimum inhibitory concentrations (MICs) of piperacillin, cefoperazone-sulbactam, piperacillin-tazobactam, cefazolin, cefuroxime, ceftazidime, ceftriaxone, ceftazidime-avibactam, cefepime, cefmetazole, aztreonam, ertapenem, imipenem, meropenem, amikacin, gentamicin, ciprofloxacin, levofloxacin, trimethoprim-sulfamethoxazole, polymyxin B, nitrofurantoin, tigecycline were determined. The MIC breakpoints for *Enterobacteriaceae* (susceptible, ≤2 mg/L; resistant, ≥8 mg/L) issued by the Food and Drug Administration were used as the breakpoints for tigecycline.

### Detection of Carbapenemase and *mcr-1* Genes

All the CRE strains were tested for the presence of the most common carbapenemase genes (*bla*_KPC_, *bla*_NDM_, *bla*_OXA-48-*like*_, *bla*_IMP_, and *bla*_VIM_) by polymerase chain reaction (PCR) with specific primers and conditions as described previously (Poirel et al., 2011; Liu et al., 2016). The colistin resistance gene *mcr-1* was also detected by PCR, as previously described (Liu et al., 2016). The positive PCR amplicons were sequenced and compared with the reported sequences from GenBank by Blast (www.ncbi.nlm.nih.gov/blast/).

### Statistical Analysis

Descriptive statistics were used to summarize the epidemiologic characteristics of CRE strains. For categorical variables, the percentage of CRE strains in each category was calculated. All analyses were performed using WHONET (version 5.6) and the IBM SPSS Statistics (version 21).

## Results

### *In vitro* Antimicrobial Susceptibility

Most of the CRE strains (>90%) were resistant to cephalosporins, piperacillin, cefoperazone-sulbactam, piperacillin-tazobactam, aztreonam, and carbapenems. Overall, 61.4, 50.1, and 45.2% of the strains were susceptible to ceftazidime-avibactam, amikacin, and trimethoprim-sulfamethoxazole, respectively, followed by gentamicin (31.8%), levofloxacin (22.9%), ciprofloxacin (19%), and nitrofurantoin (18.8%). Polymyxin B and tigecycline showed excellent antibacterial activity against CRE strains (95.8 and 98.4% susceptible, respectively) (Table 1). Ceftazidime-avibactam had potent activity against both KPC-2-producing and OXA-48-like producing *Enterobacteriaceae* (100% susceptible) and inhibited all of *bla*_KPC-2_-positive and *bla*_OXA-48-*like*_-positive strains at 8 mg/L. However, all NDM-producing *Enterobacteriaceae* were resistant to ceftazidime-avibactam (MIC_90_ > 32 mg/L). The MICs of ceftazidime-avibactam were higher than 32 mg/L against IMP- and multi-carbapenemase producing *Enterobacteriaceae* (KPC and NDM co-producers, NDM and OXA-48 co-producer). Most of the *bla*_NDM_-positive strains were susceptible to amikacin (86.2% susceptible) (Table 1).

**Table 1:** Antimicrobial susceptibility testing results of clinical CRE strains (MICs, mg/L).

**Antimicrobial agent**	**All CRE (*****n*** **=** **935)**					**KPC-producers (*****n*** **=** **482)**				**NDM-producers (*****n*** **=** **334)**				**OXA-48-like producers (*****n*** **=** **68)**			
	**MIC**	**MIC_**50**_**	**MIC_**90**_**	**%R**	**%S**	**MIC_**50**_**	**MIC_**90**_**	**%R**	**%S**	**MIC_**50**_**	**MIC_**90**_**	**%R**	**%S**	**MIC_**50**_**	**MIC_**90**_**	**%R**	**%S**
	**range**																
Piperacillin	4->256	>256	>256	98.9	0.9	>256	>256	99.4	0.2	>256	>256	99.7	0.3	>256	>256	100	0
Cefoperazone-sulbactam	1->128	>128	>128	98.3	1.2	>128	>128	98.1	1.2	>128	>128	99.4	0	>128	>128	100	0
Piperacillin-tazobactam	2->256	>256	>256	97.2	1.5	>256	>256	98.8	0.6	>256	>256	99.4	0	>256	>256	100	0
Cefazolin	32->32	>32	>32	100	0	>32	>32	100	0	>32	>32	100	0	>32	>32	100	0
Cefuroxime	2->64	>64	>64	99.9	0.1	>64	>64	100	0	>64	>64	100	0	>64	>64	100	0
Ceftazidime	0.5->32	>32	>32	98.6	0.7	>32	>32	98.1	0.8	>32	>32	99.7	0	>32	>32	100	0
Ceftriaxone	0.12-64	>32	>32	99.4	0.6	>32	>32	99.4	0.6	>32	>32	99.7	0.3	>32	>32	100	0
Ceftazidime-avibactam	0.25->32	2	>32	38.6	61.4	2	4	0	100	>32	>32	100	0	0.5	4	0	100
Cefepime	0.25->32	>32	>32	98.1	0.9	>32	>32	97.9	1	>32	>32	99.4	0	>32	>32	100	0
Cefmetazole	1->64	>64	>64	92.7	4.5	>64	>64	92.3	5.6	>64	>64	97.6	1.2	64	>64	73.5	13.2
Aztreonam	0.25->128	>128	>128	93.2	4.2	>128	>128	99	0.8	>128	>128	85.3	7.8	>128	>128	100	0
Ertapenem	0.25->32	>32	>32	98.9	1	>32	>32	99	1	>32	>32	99.7	0.3	>32	>32	100	0
Imipenem	0.12->16	>16	>16	96.1	2.1	>16	>16	99.2	0.6	16	>16	99.4	0.3	>16	>16	73.5	17.6
Meropenem	0.12->16	>16	>16	97	1.9	>16	>16	98.1	1.5	>16	>16	99.7	0.3	>16	>16	85.3	4.4
Amikacin	1->128	16	>128	49.6	50.1	>128	>128	69.7	29.9	1	>128	13.8	86.2	>128	>128	100	0
Gentamicin	1->128	128	>128	67.9	31.8	>128	>128	83.8	16	1	128	40.4	59.3	>128	>128	100	0
Ciprofloxacin	0.06->8	>8	>8	78.4	19	>8	>8	95.6	3.7	8	>8	53.6	41.3	>8	>8	100	0
Levofloxacin	0.06->16	>16	>16	76.3	22.9	>16	>16	94.6	4.8	4	>16	49.4	49.7	>16	>16	100	0
Trimethoprim- Sulfamethoxazole	0.25->32	32	>32	54.8	45.2	1	>32	47.9	52.1	>32	>32	54.5	45.5	>32	>32	100	0
Polymyxin B	0.125->16	0.25	1	4	95.8	0.25	1	4.4	95.4	0.25	1	3.6	96.1	0.5	0.5	1.5	98.5
Nitrofurantoin	4->128	>128	>128	64.1	18.8	>128	>128	92.9	4.6	64	>128	22.8	41.9	128	>128	64.7	8.8
Tigecycline	0.12–8	0.5	2	0.3	98.4	0.5	2	0.4	97.7	0.5	1	0.3	99.1	1	2	0	100

*CRE, carbapenem-resistant Enterobacteriaceae; MIC_50/90_, 50%/90% minimum inhibitory concentration; %R, % of isolates resistant; %S, % of isolates susceptible*.

### Prevalence of *bla*_KPC_, *bla*_NDM_, *bla*_OXA-48_, *bla*_IMP_, and *bla*_VIM_ Carbapenemase and *mcr-1* Genes

Carbapenemase gene was positive in 97.4% (911/935) of the CRE strains, including *bla*_KPC-2_ in 51.6% (482/935), *bla*_NDM_ in 35.7% (334/935), *bla*_OXA-48-*like*_ in 7.3% (68/935), *bla*_IMP_ in 1.7% (16/935), *bla*_KPC_ and *bla*_NDM_ in 1.0% (9/935), *bla*_NDM-24_ and *bla*_OXA-48_ in 0.1% (1/935), *bla*_NDM-1_ and *bla*_IMP-4_ in 0.1% (1/935) of the strains (Table 2). KPC-2 was the most prevalent carbapenemase among *K. pneumoniae* (64.5%, 457/709) and *S. marcescens* (100%, 8/8) strains. NDM-5 was the predominant type carbapenemase among *E. coli* (74.5%, 111/149), *E. cloacae* (66.7%, 24/36) and *C. freundii* (64.3%, 9/14). Among all OXA-48-like producing *K. pneumoniae*, PCR and DNA sequencing results showed the presence of *bla*_OXA-232_ (97.1%, 66/68) and *bla*_OXA-48_ (2.9%, 2/68) (Table 2).

**Table 2:** Prevalence of different carbapenemase genes among 935 CRE strains.

**Species**	**Strains tested, *N***	***bla*_**KPC-2**_, *n* (%)**	***bla*_**NDM**_, *n* (%)**	***bla*_**OXA-48-like**_, *n* (%)**	***bla*_**IMP**_, *n* (%)**	**Two genes, *n* (%)**	**Any gene, *n* (%)**
*K. pneumoniae*	709	457 (64.5)	*bla*_NDM-1_, 64 (9.0)	*bla*_OXA-48_, 2 (0.3)	*bla*_IMP-4_, 6 (0.8)	*bla*_KPC-2_+*bla*_NDM-1_, 6 (0.8)	693 (97.7)
			*bla*_NDM-5_, 85 (12.0)	*bla*_OXA-232_, 66 (9.3)	*bla*_IMP-69_, 3 (0.4)	*bla*_KPC-2_+*bla*_NDM-5_, 1 (0.1)	
			*bla*_NDM-3_, 1 (0.1)			*bla*_NDM-1_+*bla*_IMP-4_, 1 (0.1)	
						*bla*_NDM-24_+*bla*_OXA-48_, 1 (0.1)	
*E. coli*	149	4 (2.7)	*bla*_NDM-1_, 31 (20.8)				147 (98.7)
			*bla*_NDM-5_, 111 (74.5)				
			*bla*_NDM-3_, 1 (0.7)				
*E. cloacae*	36	3 (8.3)	*bla*_NDM-1_, 24 (66.7)		*bla*_IMP-4_, 4 (11.1)	*bla*_KPC-2_+*bla*_NDM-1_, 1 (2.8)	36 (100)
			*bla*_NDM-5_, 3 (8.3)		*bla*_IMP-6_, 1 (2.8)		
*C. freundii*	14	3 (21.4)	*bla*_NDM-1_, 9 (64.3)				12 (85.7)
*S. marcescens*	8	8 (100)					8 (100)
*E. aerogenes*	7	1 (14.3)	*bla*_NDM-1_, 1 (14.3)				3 (42.9)
			*bla*_NDM-5_, 1 (14.3)				
*K. oxytoca*	7	3 (42.9)	*bla*_NDM-1_, 2 (28.6)		*bla*_IMP-4_, 1 (14.3)	*bla*_KPC-2_+*bla*_NDM-1_, 1 (14.3)	7 (100)
*M. morganii*	3	2 (66.7)	*bla*_NDM-1_, 1 (33.3)				3 (100)
*P. vulgaris*	1	1 (100)					1 (100)
*P. rettgeri*	1				*bla*_IMP-4_, 1 (100)		1 (100)
Total	935	482 (51.6)	334 (35.7)	68 (7.3)	16 (1.7)	11 (1.2)	911 (97.4)

*CRE, carbapenem-resistant Enterobacteriaceae*.

**Figure 2:**
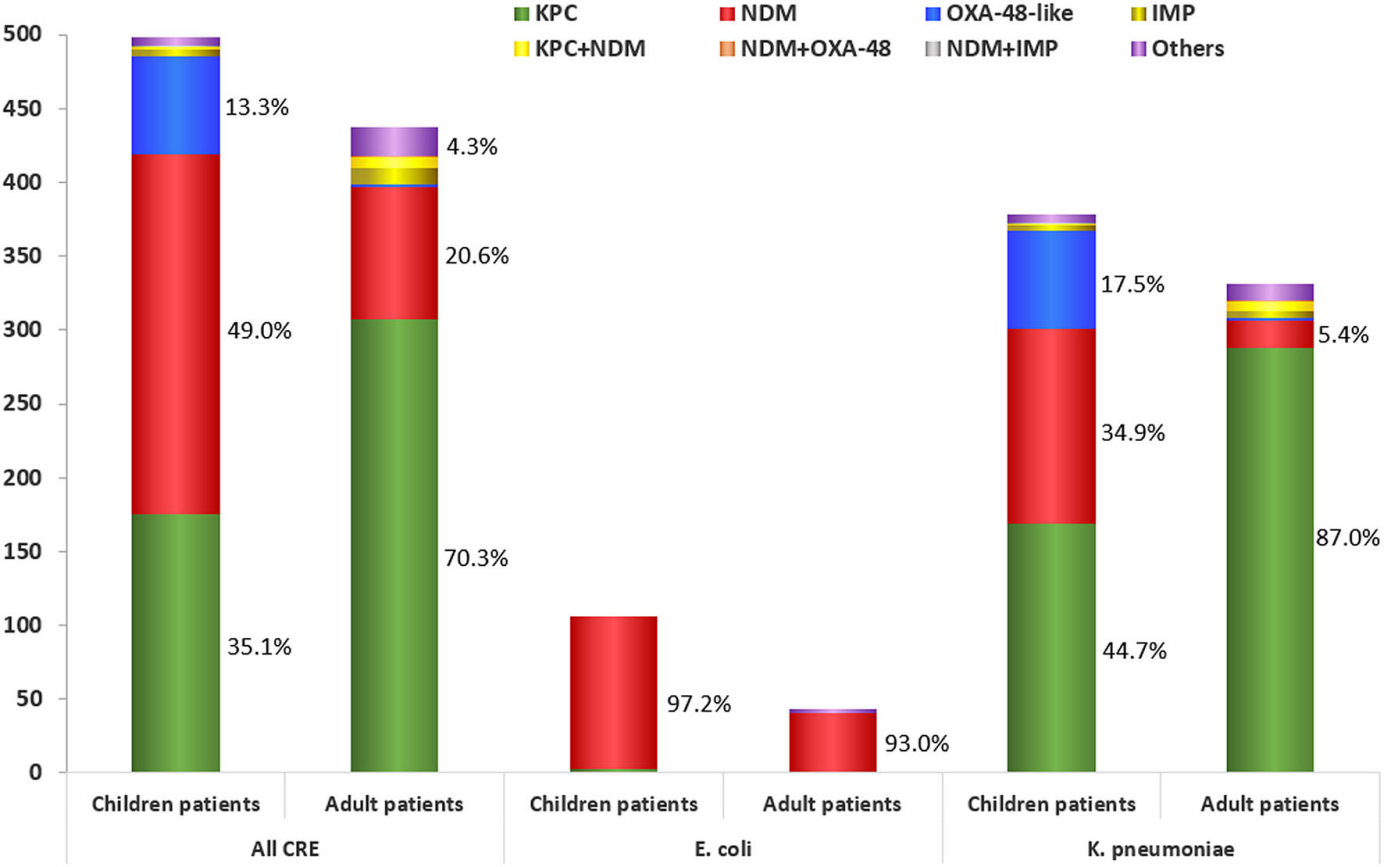
Carbapenemase distribution among the carbapenem-resistant *Enterobacteriaceae* strains isolated from adult and children patients.

Of the CRE strains isolated from adult patients, 70.3% (307/437) were KPC-2-producers; 20.6% (90/437) were NDM-producers (including 12.1% of NDM-1-producers, 8.2% of NDM-5-producers, and 0.2% of NDM-3-producer); and 0.5% (2/437) were OXA-48-producers (Table 3, Figure 2) (*P* < 0.01). However, of the CRE strains isolated from children, 49.0% (244/498) were NDM-producers (including 32.9% of NDM-5-producers, 15.9% of NDM-1-producers and 0.2% of NDM-3-producer); 35.1% (175/498) were KPC-2-producers and 13.3% (66/498) were OXA-232-producers (Table 3, Figure 2) (*P* < 0.01). The *bla*_OXA-232_-positive *K. pneumoniae* were only isolated from children patients while *bla*_OXA-48_-positive *K. pneumoniae* were isolated from adults. One polymyxin B resistant *E. coli* was positive for *mcr-1* with co-producing *bla*_NDM-5._

**Table 3:** Distribution of different carbapenemase genes in 935 CRE strains isolated from adults and children patients.

**Carbapenemase genes**	**All CRE**, ***n*** **(%)**		***E. coli***, ***n*** **(%)**		***K. pneumoniae***, ***n*** **(%)**	
	**From children**	**From adults**	**From children**	**From adults**	**From children**	**From adults**
*bla*_KPC-2_	175 (35.1)	307 (70.3)	3 (2.8)	1 (2.3)	169 (44.7)	288 (87.0)
*bla*_NDM-1_	79 (15.9)	53 (12.1)	21 (19.8)	10 (23.3)	50 (13.2)	14 (4.2)
*bla*_NDM-5_	164 (32.9)	36 (8.2)	82 (77.4)	29 (67.4)	81 (21.4)	4 (1.2)
*bla*_NDM-3_	1 (0.2)	1 (0.2)		1 (2.3)	1 (0.3)	
*bla*_OXA-48_		2 (0.5)				2 (0.6)
*bla*_OXA-232_	66 (13.3)				66 (17.5)	
*bla*_IMP-4_	3 (0.6)	9 (2.1)			2 (0.5)	4 (1.2)
*bla*_IMP-6_		1 (0.2)				
*bla*_IMP-69_	2 (0.4)	1 (0.2)			2 (0.5)	1 (0.3)
*bla*_KPC-2_+*bla*_NDM-1_	2 (0.4)	6 (1.4)			1 (0.3)	5 (1.5)
*bla*_KPC-2_+*bla*_NDM-5_		1 (0.2)				1 (0.3)
*bla*_NDM-1_+ *bla*_IMP-4_	1 (0.2)				1 (0.3)	
*bla*_NDM-24_+ *bla*_OXA-48_		1 (0.2)				1 (0.3)
Others	5 (1.0)	19 (4.3)		2 (4.7)	5 (1.3)	11 (3.3)
Total	498	437	106	43	378	331

*CRE, carbapenem-resistant Enterobacteriaceae*.

## Discussion

Previous studies have proved that the presence of carbapenemase genes, including *bla*_KPC-2_ and *bla*_NDM_, was the major mechanism of carbapenem resistance among CRE strains in China, which were the most prevalent in *K. pneumoniae* and *E. coli*, respectively (Zhang et al., 2017; Wang et al., 2018). However, the researches on CRE strains isolated from children patients are limited in China. This study provided a comprehensive and updated carbapenemase profile of 935 CRE strains isolated from both adult and children patients. We found that *bla*_KPC-2_ (51.6%) and *bla*_NDM_ (35.7%) were the most common carbapenemase genes among CRE strains, while the emergence of *bla*_OXA-232_, *bla*_IMP_, and other multi-carbapenemase genes have been increasing in recent years. KPC-2 was the most frequently detected carbapenemase gene in *K. pneumoniae*, while NDM was the most prevalent one in *E. coli*. This pattern in China is significantly different from that in Europe. In Europe, the prevalence of OXA-48-like producing *Enterobacteriaceae* was 38% (333/927), next to KPC- (42%, 393/927), but higher than NDM-producing *Enterobacteriaceae* (12%, 113/927) (Grundmann et al., 2017). The distribution of carbapenemase-producers also varied with bacterial species. In *K. pneumoniae*, KPC-producers were the most prevalent, followed by OXA-48-like (37%, 310/850) and NDM-producers (11%, 93/850). In *E. coli*, OXA-48-like producers were the most prevalent (56%, 43/77), followed by NDM- (26%, 20/77) and KPC-producers (18%, 14/77). *K. pneumoniae* and *E. coli* were the two main species in China with a ratio of 5:1 (4:1 in children, 8:1 in adults) in this study, which differed from the prevalence trends (ratio of 11:1) in EuSCAPE (Grundmann et al., 2017).

Notably, KPC-2-producers were widespread in adult patients, followed by NDM-producers, while NDM-producers were prevalent in children patients, followed by KPC-2- and OXA-48-like producers. These findings described the different patterns of carbapenemases among CRE strains from adults and children. In contrast to the previous finding that NDM-1 was the most common carbapenemase among children patients, we have found that NDM-5-producers (32.9%) were most frequently detected CRE strains from children (Tian et al., 2018; Yin et al., 2018; Zhang et al., 2018). The outbreak of NDM-5-producing ST48 *K. pneumoniae* was first reported in Shanghai (Tian et al., 2018). We speculated that outbreak of NDM-5-producers accounted for the spread of NDM-5 among children patients in this study (Tian et al., 2018; Li et al., 2020). Further study is needed to track the type of plasmids harboring these carbapenemase genes.

Unlike the previous report that few OXA-48-like producing *Enterobacteriaceae* (0.1%, 2/1801) were detected in China from 2012 to 2016 (Wang et al., 2018), we found 7.3% (68/935) OXA-48-like producing *K. pneumoniae* between 2016 and 2018. Since the first OXA-232-producing *K. pneumoniae* isolated from neonate in 2017, the outbreaks of OXA-232-producing *Enterobacteriaceae* have been successively reported in children patients (Yin et al., 2017; Tian et al., 2018). Subsequently, 10 strains of OXA-232-producing *K. pneumoniae* were isolated from elderly patients in the intensive care unit in 2019 and the *bla*_OXA-232_ was located in a 6.1-kb ColKP3-type non-conjugative plasmid, which was highly similar to the pkNICU5 first reported (similarity about 99%) in 2017 (Yin et al., 2017; Shu et al., 2019). We speculated that the presence of *bla*_OXA-232_ on a mobile element and its spread among different strains were responsible for the recent dissemination of OXA-232-producing *Enterobacteriaceae*, which would make it possible to become the “third epidemic” carbapenemase after KPC-2 and NDM in China (Yin et al., 2017; Tian et al., 2018).

All of the CRE strains were highly resistant to cephalosporins, carbapenems, aminoglycosides, and fluoroquinolones but susceptible to polymyxin B and tigecycline. Ceftazidime-avibactam, launched last year in China, showed excellent *in vitro* antibacterial activity against both KPC-2- and OXA-48-like producers, but not active against metallo-β-lactamases producers. Most (86.2%) of NDM-producers were susceptible to amikacin. In addition, we found a *bla*_NDM-5_ and *mcr-1* co-harboring *E. coli* resistant to polymyxin B. These findings limited the utility of ceftazidime-avibactam and polymyxin B and prompted the development of novel or combinational therapies to combat CRE strains. For example, aztreonam plus meropenem-vaborbactam and aztreonam plus ceftazidime-avibactam showed good antibacterial activity against NDM- and non-OXA-48-like producing *Enterobacteriaceae* (Biagi et al., 2019). The combination of colistin and amikacin showed consistently bactericidal against NDM-5-bearing *mcr-1*-positive *E. coli*, which might be an alternative therapeutic option for the treatment of lethal infections (Zhou et al., 2017).

## Conclusions

In conclusion, KPC-2, NDM, and OXA-48-like enzymes were the most prevalent carbapenemases among CRE clinical isolates in China. The most prevalent carbapenemase gene was *bla*_KPC-2_ among *K. pneumoniae* isolated from adult patients, and *bla*_NDM_ among *E. coli* isolates from both children and adult patients. The *bla*_OXA-232_ was only detected among *K. pneumoniae* isolates from children.

## Data Availability Statement

The raw data supporting the conclusions of this article will be made available by the authors, without undue reservation, to any qualified researcher.

## Ethics Statement

The study protocol was approved by the Institutional Review Board of Huashan Hospital, Fudan University (Number: 2018-408).

## Author Contributions

FH and RZ designed the study. RH, QS, SW, and MP performed the experimental work. RH and DY collected the data. FH analyzed the data. All authors read and approved the final manuscript. All authors contributed to the article and approved the submitted version.

## Conflict of Interest

The authors declare that the research was conducted in the absence of any commercial or financial relationships that could be construed as a potential conflict of interest. The reviewer YY declared a shared affiliation with one of the authors RZ to the handling editor at time of review.
